# Specialized healthcare practitioners’ challenges in performing video consultations to patients in Nordic Countries – a systematic review and narrative synthesis

**DOI:** 10.1186/s12913-022-08837-y

**Published:** 2022-11-28

**Authors:** Joanna Barbara Baluszek, Siri Wiig, Kai Victor Myrnes-Hansen, Kolbjørn Kallesten Brønnick

**Affiliations:** 1 Faculty of Health Sciences, Department of Quality and Health Technology, University of Stavanger, Stavanger, Norway; 2 Cognitive Lab: Cognitive and Behavioral Neuroscience Lab, University of Stavanger, Stavanger, Norway; 3 SHARE - Centre for Resilience in Healthcare, University of Stavanger, Stavanger, Norway; 4 Norwegian School of Hotel Management, Faculty of Social Sciences, University of Stavanger, Stavanger, Norway; 5 SESAM, Stavanger University Hospital, Stavanger, Norway

**Keywords:** Challenges, Narrative synthesis, Nordic countries, Specialized healthcare practitioners, Systematic review, Video consultations, Quality

## Abstract

**Background:**

Video consultations are becoming an important telemedicine service in Nordic countries. Its use in specialized healthcare increased significantly during COVID-19 pandemic. Despite advantages video consultations have, it may also produce challenges for practitioners. Identifying and understanding these challenges may contribute to how managers can support these practitioners and thereby improve work related wellbeing and quality of care.

**Methods:**

We designed this study as systematic review of the literature with narrative synthesis and conducted a thematic analysis. We conducted review about the use of video consultations in specialized healthcare in Nordic countries to identify and categorize challenges experienced and/or perceived by practitioners. We searched Ovid MEDLINE(R), EMBASE, APA PsycINFO, and CINAH, from 2011 to 2021. Eligibility criteria were *population* - practitioners in specialized healthcare with experience in video consultations to patients, *interest* - challenges experienced and/or perceived by practitioners and, *context* - outpatient clinics in Nordic countries.

**Results:**

We included four qualitative and one mixed method studies, published between 2018 and 2021 in Norway, Denmark, and Sweden. By thematic analysis we identified three main themes: *challenges related to video consultation, challenges related to practitioner* and, *challenges related to patient*. These themes are composed of 8 categories: *technology uncertainties, environment and surroundings, preparation for requirements, clinical judgment, time management, practitioners’ idiosyncrasies, patients’ idiosyncrasies and patients’ suitability and appropriateness.* Challenges from *technology uncertainties* category were most frequent (dominant) across all clinical specializations.

**Conclusion:**

Findings indicate the scarcity of the research and provide rationale for further research addressing challenges in providing video consultations in the Nordic context. We suggest updating this review when the amount of available research increases.

## Background

Video consultations (VCs) in healthcare are telemedicine solutions representing ‘…an approximation of face-to-face interaction and are a “visual upgrade” of widely used telephone consultations’ [1]. VCs between practitioners and patients are becoming more common in many healthcare systems worldwide, as a solution for the healthcare sector to meet the challenges of increasing referrals. At the beginning of 2020, the need for social distancing during COVID-19 pandemic has increased the use of VCs to provide medical help to patients, while preventing the spreading of the SARS CoV-2 virus among employees and patients [2].

VCs introduce geographical distance between practitioner and patient with new location and technology aspects added. These non-formal clinical settings and new circumstances, with changed professional and patient roles and responsibilities, may affect those involved, the consultation, and the outcome of the meeting. It may also introduce potential challenges such as technical problems [3, 4] concerns related to security and privacy [5] and challenges related to VCs adoption [6].

Expected and unexpected challenges during a video consultation may affect the practitioners’ behaviour, feelings, perceptions, the content, and quality of the meeting. Thus, challenges related to VCs may negatively affect the quality and safety of service [7] and thereby practitioners’ wellbeing in workplace.

It is unclear what challenges in performing VCs with patients, practitioners from specialized healthcare in Nordic countries experience and/or perceive. To our knowledge no review of this topic exists per 15th January 22. We searched the PROSPERO: International prospective register of systematic reviews using the terms: video consultations, video visit, with no success. We recognized the need and undertook a systematic review of the literature to identify, categorize, and extend evidence on challenges among Nordic specialized healthcare practitioners using VCs with patients.

We aimed to synthetize evidence to be usable for healthcare managers to better identify and understand challenges practitioners experience and/or perceive in their own workplace. Establishing this knowledge base would enable managers to better determine what areas need intervention to support acceptance, promote adoption, and prevent rejection and reluctance of VCs caused by challenges in the future, after the COVID-19 pandemic.

The research question was: what are the challenges that Nordic specialized healthcare practitioners experience when performing VCs with patients?

## Method

We designed this study as systematic review with narrative synthesis. A protocol for this study was developed by first author JBB with input from remaining authors a-priori and revised by the group iteratively. Protocol was developed accordingly to PROSPERO lay out, however was not registered in PROSPERO.

### Eligibility criteria

We applied the PICo: Population-Interest-Context [8] approach to specify eligibility criteria.

### Population

Participants in the studies had to be specialized healthcare practitioners such as doctors, nurses, psychologists, etc. By ‘specialized’ we consider healthcare practitioners based at outpatient clinics. This means that while those practitioners may have had clinical specialization, however this was not mandatory for inclusion. For example, community nurses or other staff who worked in a specialised (healthcare) team, were eligible. These who were included had to have their own experience with VCs. General practitioners in primary healthcare were not eligible. Hospital leaders, patients, next of kin were excluded, as we were interested in the perspectives of these who are directly responsible for providing VCs to patients.

### Interest

We were interested in a broad spectrum of challenges specialized healthcare practitioners perceived and/or experienced. We defined a “challenge” as a practitioner perception or as an experience of something that might limit or make it difficult to provide VCs to patients.

### Context

Studies regarding standard VCs/video visits/video conferences e.g., originated from Nordic countries and published in peer-reviewed journals were eligible. This project considers Norway, Denmark, Sweden, Finland, and Iceland as Nordic countries, as countries similar to each other (affinities). These countries also share a set of distinctive features when compared to the rest of mainland Europe, such as, for example: high life expectancy and areas with low population density. We also included autonomous Nordic territories: Faroe Islands and Greenland. Peer reviewed journal were considered as we aimed to include peer reviewed papers to avoid papers of poor quality. We considered both one off (single) consultations and follow up (control/ongoing) consultations. By standard VCs we understand conversations between practitioners and patients, with no major medical intervention. Standard VCs may differ between specializations as its requirements may differ depending on the individual progress of patients` treatment. Many specializations may require patient self-assessment of disease activity or treatment response/effect (e.g., rheumatology) or video inspection of skin changes, oedema and so on (e.g., nephrology) and these minor interventions were acceptable. Studies with quantitative, qualitative, and mixed methods designs could be included.

### Inclusion and exclusion criteria

Main inclusion and exclusion criteria of papers are presented in Table 1. Inclusion and exclusion criteria.

**Table 1:** Inclusion and exclusion criteria

**Inclusion criteria were:**
• Specialized healthcare practitioners with experience in video consultations.
• Challenges experienced and reported by the specialized healthcare practitioners.
• Outpatient clinics in Nordic countries.
**Exclusion criteria were**:
• Not about specialized healthcare practitioners.
• Challenges not found in papers.
• Not English or Norwegian language.

### Search and information sources

Two university librarians drafted and tested multiple search strings. The final search string was composed of synonyms with telemedicine, limited to five Nordic countries: Norway, Sweden, Denmark, Finland, Iceland and two autonomous Nordic territories: Faroe Islands and Greenland. We did not include terms and synonyms to challenges, practitioners, and specialized healthcare into the search string as this would limit hits and could result in missing relevant papers. Searches in databases: Ovid MEDLINE(R), EMBASE, APA PsycINFO, CINAHL were conducted on 14th July 2021. The searches were limited to 2011 - Current. The reason for the date limit is that we assume that VCs in Nordic countries were not common before 2011, and technology and technology adoption has changed a lot in the recent years, making older studies less relevant.

Detailed search strategy for Ovid MEDLINE(R) is presented in Table 2. Detailed search strategy for Ovid MEDLINE(R).

**Table 2:** Detailed search strategy for Ovid MEDLINE(R)

Database	Ovid MEDLINE(R) ALL < 1946 to July 13, 2021>	* - wildcard truncation
**Search date**	14.07.2021	
**Search** **history** **or** **procedure**	1 ((exp Telemedicine/ or (telehealth* or tele-health* or telemed* or tele-med* or ehealth* or e-health* or mhealth or m-health or mobile health or teleconsult* or tele-consult* or telenursing or tele-nursing or telepsychiatry or tele-psychiatry or remote consult*).ti,ab,kf.) and (video* or web-cam* or webcam*).mp.) or Videoconferencing/ or (((video or virtual) adj (appointment* or consult* or visit*)) or electronic consult* or econsult* or e-consult* or online consultation* or electronic visit* or e-visit* or evisit*).ti,ab,kf.	8310
	2 (norw* orswed* or denmark or danish or finland or finnish or iceland* or faroe or greenland or nordic or scandinavi*).mp,jw,lg.	584,306
	3 1 and 2	251
	4 limit 3 to yr="2011-Current"	148
**Records**	148	

### Selection of sources of evidence

The final search gave a total of 594 records: 148 records in Ovid MEDLINE(R), 195 in EMBASE, 32 in APA PsycINFO and 219 in CINAHL. A total of 594 records were exported into EndNote for screening. A total of 233 duplicates were removed, which resulted in 361 unique records. Firstly, author JBB screened titles and abstracts of all 361 papers, which resulted in inclusion of 26 papers to full text screening. After reading the full texts, four papers met the eligibility criteria and were included in the review. Author KKB undertook additional separate screening of an original EndNote file including the 594 records. This screening resulted in inclusion of 15 papers for full text screening. After reading full text, four papers were excluded, four were overlapped with already included papers by JBB and remaining seven papers were subjected to discussion between JBB, KKB and SW. From these seven papers we included two papers, then in total we decided to include six papers to review. Next JBB screened the reference lists of these six papers and found one additional eligible paper, making total of included studies seven. Grey literature search was not conducted and only papers published in peer-reviewed journals were eligible in this review. Finally, these seven papers were subjected to critical appraisal, (described detailed below in Critical appraisal of individual sources of evidence part) and two quantitative papers [9, 10] were excluded, resulting in final inclusion of five papers.

### Data items and charting process

JBB extracted the following data to Excel sheet: authors, title, country, year published, methods, participants, aim/objective, type of service, clinical specialization/condition from all the five papers. Furthermore, findings and part of context relevant to research question meaning challenges experienced and/or perceived by practitioners were extracted verbatim from the results sections in the papers. Study participants’ quotes and authors’ narratives were included. Original themes created by paper authors were excluded as that was a result of others analysis and interpretations. We did not conduct pilot testing of the data extraction, however some of data extractions were selectively respectively checked by JBB and KKB during data analysis.

### Critical appraisal of individual sources of evidence

JBB and KVHM independently appraised five qualitative studies using Checklist for Qualitative Research developed by Joanna Briggs Institute and collaborators [11]. It includes 10 questions about methodology/design with possible check boxes: yes, no, unclear, not applicable, and overall appraisal with check boxes: Include, Exclude or Seek further info. One example is “Is there congruity between the stated philosophical perspective and the research methodology? [11]. We (JBB and KVHM) met to check all 10 questions, compare results, discuss the differences and achieve consensus about all five studies. Our overall agreement/compatibility was between 60%-100%. There were some minor disagreements, for example about congruity between the research methodology and the representation and analysis of data, or about influence of the researcher on the research, and vice-versa. Overall, we achieved 100% consensus about inclusion of all those five studies.

We also carried out the same process for two quantitative studies, except that this time we used Checklist for Randomized Controlled Trials by Joanna Briggs Institute and collaborators [11] which includes 13 questions. In this case we (JBB and KVHM) achieved low agreement (15%) and were not able to achieve consensus on including or excluding those two papers. Finally, after critical appraisal (assessing eligibility and homogeneity) by KKB, those papers were excluded due to use of an undescribed questionnaire.

### Synthesis of results and analysis

We undertook narrative synthesis to organize and synthesize the data. This is an appropriate approach when data heterogeneity is large and therefore other options such as meta-ethnography is not feasible [12]. Narrative synthesis is characterized by a textual approach to the process of synthesis, relying on the use of words to describe, summarize and explain findings. This involved a preliminary synthesis in form of a thematic analysis of the individual study results. This analysis followed the basic guidance to thematic analysis suggested by Clark and Brown [13]. However, we needed to apply flexibility to our analysis to fit the research question and data. The process was composed of the following steps. JBB, after familiarization with the data set (reading and re-reading), started inductive open coding of findings. This involved naming segments of data with a label that simultaneously categorizes and summarizes each piece of data to identify what is of interest of the data. One data segment might be coded one time or multiple times. Then, the created open codes were grouped, based on similarities (sharing some characteristics). One open code might be assigned to just one group. JBB named every group thus creating eight categories. Furthermore, categories were merged in three themes. Then JBB sent the initial thematic analysis for further analytical input to KKB. The thematic analysis was later discussed between JBB and KKB. After input from KKB, JBB did a revision (recording and renaming the categories) and sent again for analytical input to KKB. JBB conducted member checking repeatedly in various phases of analysis, meaning going back to the data set at hand to see if their description (open codes) is an accurate representation [13]. The revised thematic analysis was discussed in the meeting between JBB and KKB and finally approved.

## Results

### Studies

**Fig. 1:**
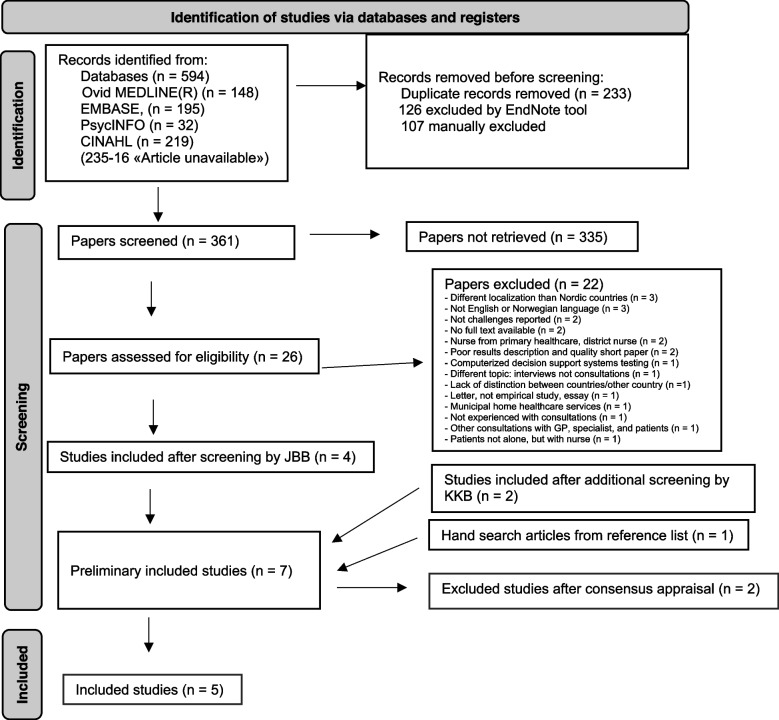
PRISMA based flow diagram [14]

We included four papers with qualitative study designs and one study with mixed method study design, published between 2018 and 2021. Two papers originated from Norway, two from Denmark and one from Sweden. One of these studies had the investigation of challenges by practitioners when providing VCs with consultation as an expressed aim. We present the process of inclusion and selection of studies based (modified) on the Preferred Reporting Items for Systematic Reviews and Meta-Analyses - PRISMA Flow Diagram [14] in Fig. 1. PRISMA based flow diagram [14].

In the study by Christensen et al. [15], the authors aimed ‘…to investigate the experiences of patients and providers regarding the use of videoconferences in older patients with depression’ [15]. They conducted a qualitative study using semi-structured interviews with patients and providers and focus group interviews with providers. They identified three main themes through thematic analysis: (1) Technical Challenges experienced by patients and providers experiences; (2) Videoconferencing as clinical supportive technology; and (3) Therapeutic relationship across face-to-face and videoconferencing formats [15].

In the study by Funderskov et al. [16], the authors aimed ‘to explore the advantages and disadvantages of using video consultations, as experienced by specialised palliative care healthcare professionals, who are involved in palliative care at home’ [16]. They conducted a qualitative study using data from field notes of an autobiographical diary, participant observations and semi-structured interviews with healthcare professionals/participants. They reported that potential barriers against using video consultations are the discussions about personal, and private issues regarding the illness, while family members are present.

In study by Sturesson et al. [17], the authors aimed to examine ‘… clinicians’ perceived limitations and disturbances, and how the conditions between patients and clinicians may change when using video visits instead of face-to-face meetings in outpatient care’ [17]. They conducted a qualitative study using observations of video visits at two different clinics and follow up interviews with clinicians. Transcripts of interviews and field notes were thematically analysed, discussed, and synthesised into themes. They reported that disturbances and limitations related to the technology were related to time; flexibility to schedule the meeting unbound of place, frustrations when the other part was late for the scheduled meeting, and that more experienced users of video visits usually waited longer before logging in. They were also related to sound; problems getting the sound to work satisfactory during the video visits, and problems with the image. Disturbances and limitations related to the surroundings were related to both the patient’s and the clinician’s environment; the principle of video technology may affect the experience and the content of the consultation, and the chosen surrounding changes the conditions for and reduces the participants’ field of view.

In the study by Tveter et al. [18], the authors aimed to investigate ‘…the experiences gained by healthcare professionals and patients’ [18] after implementation of video consultations as an alternative to hospital face-to face consultations, due to COVID-19 pandemic. They conducted a mixed method study. We focused just on the qualitative part of study, as the quantitative part was not in our interest (about patients’ experiences). The authors conducted focus-group interviews with healthcare professionals. The data from the study was sorted into categories: patient, healthcare professional, consultation, and technology [18].

In the study by Varsi et al. [19], the authors aimed to ‘*…* to investigate the perceived benefits and challenges of using video consultations in outpatient renal transplant recipient follow-up’ [19] from the perspectives of patients and health care providers. They conducted a qualitative study using semi-structured interviews with patients and providers, which were analysed using thematic analysis. They reported that the video consultation solution used in the study turned out to have major technical deficiencies. The health care providers valued the benefits provided by using video consultations but described the reoccurring technical challenges as disruptive.

In Table 3, Descriptions of the five included studies, the included studies are described.

**Table 3:** Descriptions of the five included studies

Author/ Year Country	Christensen et al. 2021 [15] Danmark	Funderskov, et al. 2019 [16] Danmark	Sturesson et al. 2018 [17] Sweden	Tveter et al. 2021 [18] Norway	Varsi et al. 2021 [19] Norway
**Aim** **of study**	‘…to investigate the experiences of patients and providers regarding the use of videoconferences in older patients with depression.’	‘To explore the advantages and disadvantages of using video consultations, as experienced by specialised palliative care healthcare professionals, who are involved in palliative care at home.’	‘… the study examines clinicians’ perceived limitations and disturbances, and how the conditions between patients and clinicians may change when using video visits instead of face-to-face meetings in outpatient care.’	‘… to investigate the healthcare professionals’ and patients’ experience of video consultations.’	‘… to investigate the perceived benefits and challenges of using video consultations in outpatient renal transplant recipient follow-up.’
**Design/** **Methods**	Semi-structured interview and focus group interview. Thematic analysis.	Field notes of an autobiographical diary, participant observations and semi-structured interviews. Systematic text condensation.	Observations of video visits and follow up in depth interviews. Thematic analysis.	Focus-group interviews. Thematic analysis.	Semi-structured interviews. Thematic analysis.
**Participants**	11 nurses and one psychologist.	Five community nurses; and three specialised palliative care team members (a head physician, a physiotherapist, and a nurse).	14 clinicians: doctors, nurses, psychologists, nutritionists, occupational therapists, and physiotherapists.	Seven rheumatologists and seven nurses.	One nephrologist and two health support personnel.
**Clinical Specialization or condition**	Psychiatry, Depression	Palliative care	Obesity	Rheumatology	Nephrology
**Service and context**	Videoconferences with patients. Conventional treatment clinics for older people with depression in a rural region of Denmark.	Video consultations with patients and between community nurses and the specialised palliative care team nurse. Patients’ homes and at the Department of Oncology, Odense University Hospital, Denmark.	Video visits with patients and patients and relatives. Outpatient care at a hospital in Sweden.	Video consultations with patients. Diakonhjemmet Hospital in Norway.	Video consultations with patients. Outpatient clinic at the Department of Nephrology at Oslo University Hospital in Norway.

#### Challenges

We identified three themes through the thematic analysis:

1. *Challenges related to video consultation*

These challenges are related to characteristics of video consultations and are represented by three categories: *technology uncertainties, environment and surroundings* and, *preparation requirements.*

*Technology uncertainties* refer to multifaceted problems with the technology not working as expected. This category was most frequent across all categories overall and appeared in all five studies. These challenges were described in studies as (among others): ‘technical problems’, ‘unstable network’, ‘video and audio problems’ [18], or ‘technology problems’ [17] or ‘technical challenges’ such as ‘… transmission interruptions and disruptions’ and ‘Dropout’ variants of Internet connectivity with informants being temporarily unable to see each other, or experiencing audio delays’ [15]. These were then open coded such as: *lack of technical functionality*, *lack of knowledge of technical issues origin, systems usability and connectivity issues, video issues, internet connection issues, insufficient media quality, poor technical devices*, and *technical deviation.*

*Environment and surroundings* refer to inappropriate and unwanted events and their consequences when the meeting is no longer at the practitioners’ office but often at patients’ home. This category appeared in two studies about obesity [17] and rheumatology [18]. These challenges were described in studies as (among others): ‘distractions in the patient’s background, for example a child sitting on the patient’s lap or playing in the same room, spouses who were doing housework...’ or patient itself who were in a store [18]. These were then open coded such as: *disturbances in patients’ surrounding and, distractions in patients’ surrounding.*

*Preparation requirements* for VCs refers to extra work that may be needed to do before, during and after VCs and do not normally occur during the traditional physical meeting (face-to face*).* This category appeared in three studies about obesity [17], rheumatology [18] and nephrology [19]. These challenges were described in studies as (among others): practitioners ‘…spent significant amount of time to make the video consultation solution work’ [19]. These were then open coded such as: *adjustment in work, availability issues, and doctor’ s extra tasks.*

2. *Challenges related to the practitioner*

These challenges are related to characteristics of healthcare practitioners and are represented by three categories: *clinical judgment, time management* and *practitioners’ idiosyncrasies*.

*Clinical judgment* refers to issues that may influence practitioners*’* perception and ability to assess while consulting patients without physical presence. This category was most frequent across challenges related to healthcare practitioners and appeared in four studies about depression [15], obesity [16], rheumatology [18] and nephrology [19]. These challenges were described in studies as (among others): ‘…they missed the opportunity to physically touch the patient and observe the environmental surroundings…’ [15], field of the view changes - ‘…patient’s surroundings provided a space that was unknown to the clinician…’ [17], …risk of overseeing (probably overlooking) important aspects [19] and ‘…lack of possibility to physically examine the patient…’ [18]. These were then open coded such as *lack of physical examination, lack of sufficient view, lack of knowledge about patients surrounding, lack of view of surroundings, lack of view of whole patient, doctors’ risk issues*, and *underreported symptoms*.

*Time management* refers to limitations related to arrangement of working time. This category appeared in one study about obesity [17]. These challenges were described in studies as (among others): delays which caused a ripple effect of delays, and thus, frustration and stress were experienced by participants. Valuable time was lost, and the meeting was shorter than planned [17]. These were then represented by open codes such as: *delays, wasted time*, and *clinicians’ poor time management.*

*Practitioners’ idiosyncrasies* refers to practitioners’ negative expectations, beliefs, prejudices, uncertainties, fears and attitudes about patients’ attitudes to perform VCs and communication skills and experience. This category appeared in three studies about depression [15], obesity [17] and rheumatology [18]. These challenges were described in studies such as (among others): practitioners’ own skepticism, prejudices and insecurity (about older patients’ technical ability) [15] and negative expectations or attitudes e.g.: ‘…suggestion that use of VCs was an economic strategy devised to increase worker productivity and reduce costs rather than on genuinely improving patient convenience and health outcomes’ [15]. These were then open coded such as: *lack of healthcare professionals’ experience/knowledge /skills, clinicians’ poor performance, negative practitioners’ attitudes about VCs purpose, negative practitioners’ attitudes about VCs use, communication/relationship issues, providers’ communication issues, empathy issues, shared negative media quality stories.*

3. *Challenges related to the patient*

These challenges are related to characteristic of patients and are represented by two categories: *patients’ idiosyncrasies* and *patients’ suitability and appropriateness.*

*Patients’ idiosyncrasies* refer to negative events caused by patients, regarding patients’ characteristics and personality. This category appeared in three studies about obesity [17], rheumatology [18] and nephrology [19]. These challenges were described as (among others): ‘…the patients lost their password…’ [19], or ‘…patient kept walking around with the video camera…’ [18]. These were then open coded such as: *frustration from patient and relative, patients’ poor performance, and possibility for sabotage of VC by patients.*

*Patients’ suitability and appropriateness* refers to these patients’ cases where the video consultation may not be reasonable. This category appeared in three studies about depression [15], palliative care [16] and rheumatology [18]. These challenges were described as (among others): ‘…complex issues and courses of therapy administered by a psychologist were considered to be less suitable…’ [15], ‘… providers agreed that use of VC was not a good approach during the acute stages…’ [15]. ‘There could be families where it could be overwhelming or degrading for the patient…’ [16] and ‘Consultations with patients who had cognitive impairments, hearing impairment, poor Norwegian language skills or needed an interpreter were deemed unsuited for video consultation’ [18]. These were then open coded such as: *lack of suitability of VCs, providers’ concerns about inappropriateness of VCs.*

Detailed description of categories and subcategories of challenges can be found in Table 4. Detailed categorized challenges in themes and frequencies in studies.

**Table 4:** Detailed categorized challenges in themes and frequencies in studies

Open codes	Category	Appearance	Frequencies	Theme
Lack of technical functionality. Lack of knowledge of technical issues origin. Systems usability and connectivity issues. Video issues. Internet connection issues. Insufficient media quality. Poor technical devices. Technical deviation.	TECHNOLOGY UNCERTAINTIES	Christensen et al. Funderskov et al. Sturesson et al. Tveter et al. Varsi et al.	5	CHALLENGES RELATED TO VIDEO CONSULTATION
Disturbances in patients’ surrounding. Distractions in patients’ surrounding.	ENVIRONMENT AND SURROUNDINGS	Sturesson et al. Tveter et al.	2	
Adjustment in work. Availability issues. Doctor’s extra tasks.	PREPARATION REQUIREMENTS	Sturesson et al. Tveter et al. Varsi et al.	3	
Lack of physical examination. Lack of sufficient view. Lack of knowledge about patients surroundings. Lack of view of surroundings. Lack of view of whole patient. Doctors’ risk issues. Underreported symptoms.	CLINICAL JUDGMENT	Christensen et al. Sturesson et al. Tveter et al. Varsi et al.	4	CHALLENGES RELATED TO PRACTICIONER
Delays. Wasted time. Clinicians’ poor time management.	TIME MENAGEMENT	Sturesson et al.	1	
Lack of healthcare professionals’ experience/knowledge /skills. Clinicians’ poor performance. Negative practitioners’ attitudes about VCs purpose. Negative practitioners’ attitudes about VCs use. Communication/relationship issues. Providers’ communication issues. Empathy issues. Shared negative media quality stories.	PRACTICIONERS’ IDIOSYNCRASIES	Christensen et al. Sturesson et al. Tveter et al.	3	
Frustration from patient and relative. Patients’ poor performance. Possibility for sabotage of VC by patients.	PATIENTS’ IDIOSYNCRASIES	Sturesson et al. Tveter et al. Varsi et al.	3	CHALLENGES RELATED TO PATIENT
Lack of suitability of VCs. Providers’ concerns about inappropriateness of VCs.	PATIENTS’ SUITABILITY AND APPROPRIATENESS	Christensen et al. Funderskov et al. Tveter et al.	3	

## Discussion

We conducted a systematic review of the literature with narrative synthesis to identify and categorize challenges Nordic specialized healthcare practitioners experience and perceive when performing VCs with patients. We included only five papers in this review, which suggests scarcity of research designed to investigate challenges related to use of VCs from specialized healthcare practitioners’ perspective. We did not find papers originating from Finland or Iceland, neither from Greenland nor Faroe Islands. Only one eligible paper investigated challenges related to the use of VCs as the expressed aim [19]. Other papers had investigation of *experiences* [15, 18], *disadvantages* [16] *limitations and disturbances* [17] as expressed aims. The studies spanned five clinical specializations such as rheumatology, nephrology, obesity, psychiatry, and palliative care. We did not find evidence of published papers about other clinical specializations which highlights a gap in the research.

We identified three main themes through thematic analysis: *(1) Challenges related to consultation; (2) Challenges related to practitioner;* and *(3) Challenges related to patient.* These themes were composed of 8 categories such as: *technology uncertainties, environment and surroundings, preparation for requirements, clinical judgment, time management, practitioners’ idiosyncrasies*, *patients’ idiosyncrasies;* and *patients’ suitability and appropriateness.*

Our findings indicate that challenges from the *technology uncertainties* category are dominant as they were reported in all five studies, thus being present across all clinical specializations. The reason for this high prevalence may be potentially explained by Sturesson et al. (2018) who concluded that there is a change in roles during video visits (consultations) in a form where the clinician (practitioner) assumes the responsibility of providing first line support if both (s)he and the patient encounter problems with the technology [17]. Another possible explanation is that the occurrence of challenges depends on what kind of technology solutions are used. Video communication technology should be reliable, user-friendly, suitable, and tailored to user both in healthcare settings and home settings, but this is not always the case. Furthermore, lack of or insufficient training may worsen the impact of these challenges. For example, *Video issues* during VCs may cause the clinician’s attention to be drawn from medical issues and over to the technical issues, and thus the content of the meeting is changed. Other authors also suggest that the occurrence of technological problems and inadequate device quality associated with the use of information and communication technologies may lead to the discontinuation or abandonment of telecare services [7]. It is interesting therefore that practitioners’ attitudes, expectations, and opinions, both negative and positive, may influence how they perceive and experience technology uncertainties. In the study by Christiansen et al. (2021) the authors noticed that challenges related to technical uncertainties were especially highlighted by providers with negative attitudes and expectations [15]. At the same time, it seems that the strength of positive attitudes related to use of videoconferences counteracted the negative technical experiences. This increased the adoption of videoconferencing as a tool for clinical support and enabled the development of a therapeutic relationship using videoconferencing [15]. What is more, in the study by Varsi et al. (2021) the authors concluded that benefits from using VCs as an alternative to in-person consultations may outweigh potential technological challenges for health care providers [19].

We noticed that some challenges were common across clinical specializations, suggesting that there are universal issues that can be addressed when practicing VCs with patients in outpatient clinic. On the other hand, some challenges appeared to be specific for specializations or conditions, as clinical specializations or conditions may vary in tasks which needed to be done before consultation. Example here might be rheumatology, where the lack of blood test done before consultation was noticed as drawback [18]. In the study of Christensen et al. (2021) about psychiatry clinical specializations, no challenges from the category of *challenges related to the patients*, were found. In contrast, in the study of Tveter et al. (2021) in rheumatology clinical specializations, there were most challenges from the category of *challenges related to the patients*, reported. This variation in challenges suggests that conditions and environment differ across specializations, for example some specializations require more material preparation (rheumatology) [18], than others (psychiatry) [15]. This difference suggests the importance of considering the context for VCs, particularly the importance of considering challenges that may be specific for given specialization, when planning for VCs.

Challenges accompanying VCs may be a burden to practitioners and thus have negative impact on emotional wellbeing among practitioners and hence on quality and safety of their service. The impact of the patient’s environment and surroundings, while in VCs, may introduce challenges which may also affect the communication [20] outcome of the consultation, and work performances during the meeting [21, 22]. Challenges from the *clinical judgment* category were the second most common challenges across all clinical specializations in our review. The ability to do sufficient clinical judgment is essential for practitioners. Lack of possibility for physical examination of patients during VCs may cause fear of not having (enough) adequate health information about patients, of not detecting those who underreport their symptoms and setting the wrong diagnosis [18]. Therefore, healthcare leaders aiming for resilience in healthcare when introducing VCs (and constantly thereafter during daily practice) as a service for patients in clinics, should reduce the possibility for occurrence of challenges while practicing VCs. Furthermore, having adequate measures when such challenges occur to support practitioners, is recommended. When practitioners experience a feeling of not capturing the whole picture of patient`s condition, it should be possible to reschedule a video consultation to a physical visit. Undoubtedly, VCs are not appropriate or suitable for all patients and careful choice of the patients eligible is essential to maintain good quality VCs. VCs cannot replace all face-to-face conversations. Videoconferences, in most cases, were best suited for shorter follow-up consultations, for stable patients with no confounding issues [18, 19] as patients did not require physical examination [19].

Our review has some potential limitations. First, this review does not include ongoing research. There is possibility for not identifying whole spectrum of challenges. For example, previous research, both from non-Nordic countries and Nordic countries raise concerns about security and privacy; expressed from patients’ perspectives [5]. Also, in the study by Varsi et al. (2021) are these concerns mentioned. Issues of security and confidentiality are reported by patients but not by the healthcare personnel. This does not necessarily mean that these issues are not seen or perceived as potential challenges by the practitioners. These were however not identified in our review.

The other limitation is papers’ eligibility issue. We included papers conducted on practitioners from specialized healthcare in Nordic context. This led to the exclusion of challenges seen in non-Nordic context - which might potentially bring broader understanding of the study topic. Furthermore, we included some papers with the review’s inclusion and exclusion criteria, which were not 100% eligible. For example, in one study, not all practitioners had personal experience in providing VCs [15]. This means that some of them were observers. It was not possible to distinguish in the original paper whether the mentioned challenges came from the experienced practitioners or inexperienced ones. Ordinarily we wanted to include just the challenges experienced and reported by the specialized healthcare practitioners (reported perceptions of just experienced practitioners). This was not feasible due to lack of sufficient descriptions or clarity of some of the narrative in papers. For example, some authors reported observations and citations from practitioners, patients, and themselves together, for example by writing: ‘most patients and providers' [15–17]. It was therefore difficult to judge who said/perceived/experienced what. Furthermore, challenges that occurred in one study were not always perceived or recognized by practitioners from specialized healthcare [16]. In addition, it was difficult to distinguish in one of the papers if the observation was about problems with IT solutions (Skype) or not. We found just one study aiming explicitly at identifying challenges with VCs. Thus, as the challenges were not directly named as challenges, we needed to identify them, carefully reading between the lines. Moreover, despite poor data availability (lack of citations from practitioners) or no transparent results (e.g., tables) available, we did not attempt to contact authors to obtain raw data. We also admit the possibility of missing of some important papers or challenges, although we used comprehensive search string and more than four databases were searched. Furthermore, the hand search in literature list was done by one person and grey literature search was not conducted - as we wanted to include just peer-reviewed papers to avoid papers of poor quality.

Another limitation is advanced data heterogeneity. It was difficult to form a coherent picture of the challenges, due to significant heterogeneity of data, with variations in study designs, methods, study participants, context, and clinical specializations. This might impact consistency of analysis and interpretation of results.

Thematic analysis was a matter of our (JBB and KKB) interpretation. The process of inductive open coding of findings from included studies, was a subjective process to reduce the amount of text. This process might be influenced by the fact that we searched and read papers on the topic before the analysis. This might produce/create prejudices and coding might be therefore a combination of inductive and deductive approach. Furthermore, construction of descriptive themes based on these codes, and according to the research question, was also a subjective process. All the above might potentially influence consistency and coherency of analyses. SW and KVMH have both however critically read this paper, the codes, categories and themes, and their related descriptions, evaluating the outcomes and thus strengthening the level of credibility.

## Conclusion

As the recent times, especially the COVID-19 pandemic shows, the changes and an increased use of VCs is currently happening. Identification and understanding of challenges are important for the relevant stakeholders, who address and are responsible for key areas such as quality of service, employees’ mental health, and occupational health. Therefore, this review takes on the task to gather and analyse the evidence, which is available, already today. Furthermore, this review aims to aid in the development of future research and hoping it will be of interest to any researcher planning similar review, to allow for future improvements. The ultimate goal of this paper is to provide a basis, directly or indirectly, to support practitioners in their quest to provide VCs to a wide group of patients; and to allow for additional training and/or interventions to improve performance and quality. We conclude after conducting this review that challenges from *technology uncertainties* category are dominant. However, our findings indicate the scarcity of the research and provide rationale for further research addressing challenges in providing video consultations in the Nordic context. We suggest updating this review when the amount of available research increases.

## Data Availability

All included studies are publicly available and can be found from the references of this paper.

## Abbreviations

VCs: Video consultations
